# Public Opinions and Conspiracy Thinking Toward the COVID-19 Outbreak in Iraqi Kurdistan

**DOI:** 10.1017/dmp.2020.490

**Published:** 2020-12-22

**Authors:** Deldar Morad Abdulah, Mahir Sadullah Saeed

**Affiliations:** 1 Community and Maternity Health Nursing Unit, College of Nursing, University of Duhok-Iraqi Kurdistan, Duhok, Iraq; 2 Emergency Department, Azadi Teaching Hospital, Duhok General Directorate of Health, Iraqi Kurdistan, Duhok, Iraq

**Keywords:** COVID-19 outbreak, conspiracy thinking, belief

## Abstract

****Objective**:**

The public’s perceptions toward the COVID-19 crisis and the government’s attempts to handle the crisis are critically noteworthy. The public opinions toward the COVID-19 crisis were explored in this study.

****Methods**:**

In this report, 1102 participants were included from 2 popular social media platforms from the Duhok Governorate in Iraqi Kurdistan between June 2 and 22, 2020, through an online technique.

****Results**:**

The study revealed that 14.0% of the participants believed that there is no COVID-19 in this region, and 20.1% had no concerns about the disease spread. This study revealed that 27.4% had conspiracy thinking about the COVID-19 outbreak, including that the outbreak is a plot against/of the Kurdistan Region Government, 16.4% and 19.3%, respectively. The outbreak caused considerable changes in participants’ lives (85.8%). The participants who had conspiracy thinking were younger (27.0 vs 30.0; *P* = 0.001) and had a higher level of education (37.50% high school and under, 26.0% college and above, 16.2% illiterate: *P* < 0.001). In addition, they had a private job (43.7%), and were unemployed (23.9%; *P* < 0.001), and had negative views on the TV information (38.9% vs 17.5%; *P* < 0.001).

****Conclusions**:**

A considerable percentage of the public exhibits conspiracy thinking toward the COVID-19 crisis in Iraqi Kurdistan.

An epidemic of cases of unexplained low respiratory infections was detected in China in 2019. The virus is very contagious and spreads quickly globally. The outbreak of the novel coronavirus disease 2019 (COVID-19) was declared by the World Health Organization (WHO) on January 30, 2020. The outbreak has spread to several countries,^1^ including Iraqi Kurdistan.^2^

The virus primarily spreads by means of respiratory droplets between persons who have close contact with each other. These respiratory droplets are produced through the coughing and sneezing of an infected person. A person may also be infected with COVID-19 by touching a surface or object contaminated with the live virus.^3^ Governments across the world are creating countermeasures to alleviate the possible devastating effects of this outbreak.^1^ The Kurdistan Region’s Ministry of Health announced on Friday, February 25, 2020, that they are taking all required measures to prevent this new fatal virus from spreading within the autonomous Kurdistan Region of Iraq. The Kurdistan Regional Government (KRG) has put together several new precautionary measures and recommendations to prevent the COVID-19 spread from Iran to the Kurdistan Region. By July 23, 2020, a total of 11,571 confirmed cases were reported in Iraqi Kurdistan; including recovered and dead individuals 6309 and 444, respectively.^4^

The public’s perceptions of the COVID-19 crisis are critically noteworthy, because their perceptions affect the overall adherence to preventive measures. If the public is sceptical about the legitimacy of the crisis or the government’s actions, their cooperation could begin to decline. The general public is important to the success of health and preventive measures.

Social media has become the main platform for the public to present their opinions and beliefs about different medical and nonmedical issues. Web-based social and communication technologies are widely used by the public. In this regard, we explored the public beliefs of Duhok governorate toward the COVID-19 outbreak in Iraqi Kurdistan in this survey.

## Participants and Methods

### Study Design and Sampling

Persons who live in different geographic areas of the Duhok Governorate in Iraqi Kurdistan were invited in this survey. The individuals were invited through an online technique to avoid disease spread. Few individuals visit public areas during the COVID-19 outbreak in this region. In addition, the government has considerably reduced the business hours of governmental organizations during the COVID-19 outbreak. Therefore, we created an online Google form to collect the required information from the public.

The authors collected information from the main social media platforms. Facebook and Viber are the most popular social media platforms in this region. The participants were invited from several Facebook pages and groups. The authors attempted to invite different social media groups, such as employee groups, medical doctors, nurses, businessmen, market pages, etc. We encouraged participants to send the form to their family members to include individuals with different attitudes and education levels.

Many individuals communicate through social media owing to social distancing and movement restrictions. They communicate, share, and express their perceptions and experiences surrounding COVID-19 by means of social media. Therefore, the best way to collect the information was by creating a Web-based questionnaire and sending it through social media. We tried to include individuals with different social circles between June 2 and 22, 2020.

### Study Setting

The Kurdistan region of Iraq has 4 official governorates: Erbil, Sulaymaniyah, Halabja, and Duhok. On March 1, 2020, the first cases of COVID-19 were announced in the Sulaymaniyah Governorate. The first cases were a family and women who had just returned from Iran.

### Inclusion and Exclusion Criteria

Adult persons of both genders living in different geographic areas of the Duhok Governorate, irrespective of socio-demographic aspects, were eligible to participate in this survey. The participants had different education levels, occupations, and religions. Also, individuals had to be able to answer the items in the questionnaire to be eligible for participation. We invited many individuals from different locations to obtain a representative sample of the region. The participants had to respond to all questions to submit the Google form; therefore, there was no information missing from this study. Finally, 1102 participants were included in this study.

### Data Collection and Measures

In the first part of the questionnaire, the authors collected some general information, including their age, gender, and level of education (categorized as illiterate, high school, and under or college graduate). Each participant’s place of work was categorized as work in the Ministry of Health, other governmental organizations than health, private-sector job, or unemployed.

The second part of the questionnaire had 12 binary questions about beliefs toward the COVID-19 outbreak. The questions were obtained from the literature.^5^ We asked the participants the following questions: “Do you believe that there is COVID-19 in this region?”, “Are you concerned about the spread of COVID-19 infection?”, “Do you follow news of the COVID-19 outbreak in Kurdistan?” and “Do you consider the information on TV and social media about COVID-19 positive?”

Moreover, we asked each participant whether they believe that COVID-19 is a plot against the Kurdistan Region and whether they believe that COVID-19 is a plot of the Kurdistan Region Government. We also asked them if their lives had changed since the onset of COVID-19 in Kurdistan, whether they expect the crisis to be solved within a few more months, and whether they trust the Kurdistan Government to handle the outbreak. We also asked each participant whether they are ready to follow further instructions from the government, and if they are tired to extend the curfew in Iraqi Kurdistan.

### Statistical Analysis

The ages of the participants are presented in terms of median and interquartile range. The age groups and other general information were presented in terms of number and percentage. The public beliefs toward the COVID-19 outbreak were presented in terms of number and percentage. The association of participants’ characteristics with public beliefs toward the COVID-19 outbreak was examined in Mann-Whitney U-test or chi-squared test. A *P*-value of less than 0.05 was considered statistically significant. The statistical analyses were performed using Statistical Package for the Social Sciences Version 25 (IBM SPSS Statistics for Windows, Version 25.0. Armonk, NY: IBM Corp).

## Results

The median age of the participants was 29.0 y (range, 16-82 y) and were males (70.7%) and females (29.3%). The participants had various educational levels and worked in different locations (see Table 2).

Of the participants, 14.0% believed that there is no COVID-19 in this region, and 20.1% had no concerns about the spread of the disease. The participants reported that they did not follow news of the COVID-19 outbreak in Kurdistan (18.7%) and believe that TV news (46.5%) and social media (65.5%) play a negative/stressful role. The prevalence of conspiracy thinking in respondents was 27.4%. The study found that 16.4% reported that COVID-19 is a plot against the Kurdistan Region, whereas 19.3% reported that COVID-19 is a plot of the KRG. A considerable percentage of the participants reported that the COVID-19 outbreak has changed their lives (85.8%). A considerable percentage of the participants reported that they were tired of the curfew in Iraqi Kurdistan (58.0%) (Table 1).

**Table 1.** Public beliefs toward COVID-19 outbreak

Beliefs (*N* = 1102)	Statistics	
	Yes	No
Do you believe that there is COVID-19 in this region?	948 (86.0)	154 (14.0)
Do you have concerns about the spread of COVID-19 infection?	881 (79.9)	221 (20.1)
Do you follow the news of the COVID-19 outbreak in Kurdistan?	896 (81.3)	206 (18.7)
Do you consider the positive the information on TVs about the COVID-19?	590 (53.5)	512 (46.5)
Do you consider the positive the information on social media about the COVID-19?	380 (34.5)	722 (65.5)
Do you think that COVID-19 is a plot against Kurdistan Region?	181 (16.4)	921 (83.6)
Do you think that COVID-19 is a plot of the Kurdistan Region?	213 (19.3)	889 (80.7)
Has the virus changed your lives since its onset in Kurdistan?	945 (85.8)	157 (14.2)
Do you expect the crisis to be improved within a few more months?	545 (49.5)	557 (50.5)
Do you trust in the Kurdistan government to handle the outbreak of the COVID-19?	538 (48.8)	564 (51.2)
Are you ready to follow further instructions of the government?	938 (85.1)	164 (14.9)
I am tired of extending the curfew in Iraqi Kurdistan.	639 (58.0)	463 (42.0)
Conspiracy thinking (the outbreak is a plot of/against the KRG)	302 (27.4)	800 (72.6)

The participants who had conspiracy thinking were younger (27.0 vs 30.0 y; *P* = 0.001), had a higher level of education, and had private-sector jobs (43.7%) or were unemployed (23.9%) *P* < 0.001). The participants who held a negative view of the information about COVID-19 provided by means of TV were more likely to have conspiracy thinking about the COVID-19 outbreak (38.9% vs 17.5%; *P* < 0.001) (Table 2).

**Table 2.** Association of participants’ characteristics with public beliefs toward COVID-19 outbreak

Characteristics (*N* = 1102)	Total	Having conspiracy thinking		P-Value
		Yes	No	
Age (range: 16-82 y)	29 (12)	27.0 (11.0)	30.0 (12.0)	0.001^a^
Age groups				0.016^b^
16-19	65 (5.9)	19 (29.2)	46 (70.8)	
20-29	516 (46.8)	163 (31.6)	353 (68.4)	
30-39	377 (34.2)	84 (22.3)	293 (77.7)	
40-49	106 (9.6)	23 (21.7)	83 (78.3)	
50 and over	38 (3.4)	13 (34.2)	25 (65.8)	
Gender				0.334^b^
Male	779 (70.7)	220 (28.2)	559 (71.8)	
Female	323 (29.3)	82 (25.4)	241 (74.6)	
Education				<0.001^b^
Illiterate	74 (6.7)	12 (16.2)	62 (83.8)	
High school and under	200 (18.1)	75 (37.5)	125 (62.5)	
College and above	828 (75.1)	215 (26.0)	613 (74.0)	
Occupation				<0.001^b^
Ministry of Health	306 (27.8)	73 (23.9)	233 (76.1)	
Other Ministries	283 (25.7)	70 (24.7)	213 (75.3)	
Private job	183 (16.6)	80 (43.7)	103 (56.3)	
Unemployed	330 (29.9)	79 (23.9)	251 (76.1)	
Do you consider the positive the information of TVs about COVID-19?				<0.001^b^
Yes	590 (53.5)	103 (17.5)	487 (82.5)	
No	512 (46.5)	199 (38.9)	313 (61.1)	
Do you trust in the Kurdistan government to handle the outbreak of the COVID-19?				0.069^b^
Yes	538 (48.8)	134 (24.9)	404 (75.1)	
No	564 (51.2)	168 (29.8)	396 (70.2)	

a Mann-Whitney U-test was performed for statistical analyses.

b Pearson chi-squared test was performed for statistical analyses.

## Discussion

This study shows that a considerable percentage of a sample of the public in Iraqi Kurdistan have conspiracy thinking about the COVID-19 outbreak. The surveys conducted across the world show that the public is concerned about the spread of COVID-19. A Gallup survey shows that 63.0% of Americans are very worried or worried about being exposed to the COVID-19 and 93.0% follow the crisis very closely or somewhat closely.^6^ The survey reported that the public remains relatively skeptical of the news media, and only 44.0% approve of the way the media are handling the situation.^6^

Abacus Data Bulletins reported that 75% of Canadians follow news or information on the COVID-19 outbreak either closely or very closely. The survey reported that 69% worry and 40% worry a lot or are extremely worried. Of interest, 37% reported that they have visited a public health website about COVID-19, and millions have visited a pharmacist, health professional, or clinic.^7^ Americans have reported that the COVID-19 outbreak poses a major threat to their health, population, and economy in terms of job loss, pay cuts, and reduced working hours.^5^

Chamier, Noel et al.^8^ reported that the COVID-19 outbreak has had a positive effect on trust in governments despite the disruption caused by the pandemic. The increase in public trust in governments establishes a golden opportunity to take decisive action concerning the outbreak. However, it must be taken into account that this trust may not last, particularly if the response is ineffective. The public across the globe has mentioned that there is an urgent need to reopen the economy, even with the public health risks associated with ending the lockdown.

We found that participants with a higher level of education, participants with private-sector jobs, and unemployed participants were more likely to have conspiracy thinking. In our opinion, this response is a reaction unique to the participants rather than a reflection of majority public belief. In further examination, we found that the participants with a higher level of education were more likely to have a private-sector job or to be unemployed (data not shown in tables). We believe that the participants with private-sector jobs have been affected remarkably by the COVID-19 outbreak during the lockdown and social distancing policies in this region. The majority of them reported that their lives have been changed since the onset of the COVID-19 outbreak in Kurdistan. Fernandes^9^ discussed that severe lockdowns resulted in a reduction in consumption and interruption to production. The functioning of global supply chains has been disrupted, affecting companies all over the world. In addition, millions of individuals have lost their jobs due to company shutdowns.

We do not have official information about the effect of lockdown and social distancing strategies on the financial status of individuals with private or small businesses. However, it seems that these businesses have been affected as other countries across the world.^10^ Also, they reported that their lives have been changed since the pandemic onset in Kurdistan. We do not know the exact reasons for the conspiracy thinking about the COVID-19 pandemic. It may reflect the kinds of conspiracy theories and pseudoscientific claims. The high levels of conspiracy thinking in the public result in the rejection of scientific findings.^11^ In addition, the percentage reported in this study may not be representative of all Kurdish population in Iraq, because we collected the information in 1 governorate only.

### Limitations

The findings reported in this study must be reported with caution, particularly because social distancing and lockdowns did not allow us to collect the information through face-to-face interviews. In addition, the low percentage of illiterate individuals in this study may reflect the low participation of the illiterate public in social media. Importantly, the sample taken for this study may not be representative of all Kurdish populations in this region. The current situation did not allow us to reach the information from other governorates in Iraqi Kurdistan.

## Conclusions

This study illuminates public beliefs and concerns about the presence and the spread of COVID-19 in 1 governorate in Iraqi Kurdistan. A noticeable percentage of the participants have conspiracy thinking about the COVID-19. A considerable percentage of the participants who had conspiracy thinking were unemployed, had higher-level education, had private-sector jobs, or had negative views of TV information about the COVID-19 outbreak. More than half of the participants do not trust the Kurdistan Government to handle the COVID-19 crisis within the next few months, but they are ready to follow further instructions from the government.
